# Essential oils block cellular entry of SARS-CoV-2 delta variant

**DOI:** 10.1038/s41598-022-25342-8

**Published:** 2022-11-30

**Authors:** Luiz Torres Neto, Maria Lúcia Guerra Monteiro, José Fernández-Romero, Natalia Teleshova, James Sailer, Carlos Adam Conte Junior

**Affiliations:** 1 Center for Food Analysis (NAL), Technological Development Support Laboratory (LADETEC), Cidade Universitária, Rio de Janeiro, RJ 21941-598 Brazil; 2 Laboratory of Advanced Analysis in Biochemistry and Molecular Biology (LAABBM), Department of Biochemistry, Federal University of Rio de Janeiro (UFRJ), Cidade Universitária, Rio de Janeiro, RJ 21941-909 Brazil; 3 Graduate Program in Food Science (PPGCAL), Institute of Chemistry (IQ), Federal University of Rio de Janeiro (UFRJ), Cidade Universitária, Avenida Athos da Silveira Ramos, N. 149, Bloco A, 5° Andar, Rio de Janeiro, RJ 21941-909 Brazil; 4 Graduate Program in Veterinary Hygiene (PPGHV), Faculty of Veterinary Medicine, Fluminense Federal University (UFF), Vital Brazil Filho, Niterói, RJ 24220-000 Brazil; 5 Science Department, Borough of Manhattan Community College, The City University of New York, 199 Chambers Street, Science Department Room N699, New York, NY 10007 USA; 6 Center for Biomedical Research, Population Council, 1230 York Avenue, New York, NY 10065 USA; 7 Graduate Program in Sanitary Surveillance (PPGVS), National Institute of Health Quality Control (INCQS), Oswaldo Cruz Foundation (FIOCRUZ), Rio de Janeiro, RJ 21040-900 Brazil

**Keywords:** SARS-CoV-2, Mass spectrometry, Natural products

## Abstract

Aiming to fill a gap in the literature, we aimed to identify the most promising EOs blocking in vitro cellular entry of SARS-CoV-2 delta variant without conferring human cytotoxicity and provide insights into the influence of their composition on these activities. Twelve EOs were characterized by gas chromatography coupled to mass spectrometry. The antiviral and cytotoxicity activities were determined using the cell-based pseudoviral entry with SARS-CoV-2 delta pseudovirus and the XTT assay in HeLa cells expressing human angiotensin-converting enzyme 2 (HeLa ACE-2), respectively. *Syzygium aromaticum*, *Cymbopogon citratus*, *Citrus limon*, *Pelargonium graveolens*, *Origanum vulgare*, “*Illicium verum*”, and *Matricaria recutita* showed EC_50_ lowered or close to 1 µg/mL but also the lowest CC_50_ (0.20–1.70 µg/mL), except “*I. verum*” (30.00 µg/mL). Among these, “*I. verum*”, *C. limon*, *P. graveolens* and *S. aromaticum* proved to be promising alternatives for SARS-CoV-2 delta variant inhibition (therapeutic index above 4), which possibly was related to the compounds (E)-anetole, limonene and beta-pinene, citronellol, and eugenol, respectively.

## Introduction

Since the emergence of the COVID-19 pandemic, numerous measures to control this virus's spreading have been taken worldwide^1^. Although several countries are vaccinating their population, the pandemic is not over yet^2^, and the treatment of COVID-19 is still a real challenge^3–5^, mainly due to the new emerging variants.

The emergence of new variants contributes to the continued global circulation of SARS-CoV-2, and they are classified as variants of concern (VOCs) due to their easy transmission^6^. The VOCs have modifications in the spike protein and thus important implications for transmission rate, disease severity, and immune evasion, such as Omicron and, mainly, the Delta variant for its high infectivity^7,8^. Furthermore, the neutralizing antibodies produced from first-generation vaccination or natural infection with COVID-19 may not bind with the same affinity to VOCs^9,10^. Synthetic drugs such as chloroquine, hydroxychloroquine, and molnupiravir have been suggested as potential treatments against COVID-19. However, these drugs are doubtful in terms of effectiveness versus side effects and toxicity^11^, highlighting the need for continuous research to discover new natural alternatives for the treatment, control, and inactivation of the SARS-CoV-2 virus.

Natural compounds have been studied as potential antiviral agents against SARS-CoV-2 viruses^5,12–15^. Among the secondary metabolism products of plants, essential oils (EOs) have shown promising antiviral activity against several viruses^16^. For this reason, these oils have been considered a potential source of bioactive compounds for impairing SARS-CoV-2 replication or supporting the treatment of some symptoms of COVID-19^17,18^.

In the last two years, numerous review articles have hypothesized an effective action of EOs against SARS-CoV-2 based on their activity against other viruses such as human immunodeficiency virus (HIV), influenza A virus (H1N1), Zika virus, human herpes virus (HSV) and, avian influenza A virus (H5N1) and among others^16,19–23^. Similarly, in silico studies have addressed a high binding affinity of numerous subclasses of terpenes and sesquiterpenes in the main target proteins of the SARS-CoV-2 virus, such as main protease (M_pro_/3CL_pro_), spike protein (S_pro_), angiotensin-converting enzyme-2 (ACE2), enzyme transmembrane protease serine-type 2 (TMPRSS2), RNA-dependent RNA polymerase, and others^18,24–28^. Beyond the favorable binding affinity of EOs on SARS-CoV-2 target proteins, in silico studies have demonstrated that their bioactive compounds have satisfactory pharmacokinetic and toxicity characteristics concerning absorption, distribution, metabolism, excretion, and toxicity^18^.

Nevertheless, despite this fact, there are only two in vitro studies with this approach to date. Senthil Kumar et al.^29^ observed significant ACE2 inhibitory effects in epithelial cells by *Pelargonium graveolens* and *Citrus limon* EOs, indicating a possible downregulating activity in the ACE2 receptor. These authors also reported successful results of both EOs for cytotoxicity in HT-29 cells. Ćavar Zeljković et al.^12^ evaluated the anti-SARS-CoV-2 activity of essential oils from Lamiaceae plant species and their monoterpenes and reported a notable activity for carvone, carvacrol, pulegone, menthofuran, and 1,8-cineole. According to these authors, while carvacrol and menthofuran presented good cytotoxicity, carvone, pulegone, and 1,8-cineole showed no activity in Vero 76 cells. In vitro studies are essential to validate in silico results, understand the real anti-SARS-CoV-2 potential of EOs, and boost in vivo studies for further commercial use.

We recently published a systematic and meta-analytic review from in silico studies, which aimed to identify the most EO promising molecules among more than 400 compounds and EO potential sources against SARS-CoV-2 based on their binding affinity by target proteins, pharmacokinetic, and toxicity properties^18^. Furthermore, EOs with potential antiviral activity against other viruses as described in the literature were also considered in this study. Therefore, this study aimed to assess the antiviral actitvity and cytotoxicity of twelve species of EOs against the SARS-CoV-2 delta variant. A chemometric approach was used to provide insights into the influence of EOs composition on their activity, which represents another area that needs more investigation.

## Results and discussion

EC_50_, CC_50_, and TI values are shown in Table 1. The antiviral EC_50_ values varied from 0.09 to 57.1 µg/mL, revealing that the SARS-CoV-2 Delta variant was susceptible to all EOs tested. In a descending order of antiviral activity, the most active were *S. aromaticum* (0.09 µg/mL), *C. citratus* (0.1 µg/mL), *C. limon* (0.15 µg/mL), *P. graveolens* (0.2 µg/mL), and “*I. verum”* (0.5 µg/mL).

**Table 1:** In vitro antiviral and cytotoxic activities of twelve essential oils expressed in half-maximal effective concentration (EC_50_), half-maximal cytotoxic concentration (CC_50_), and therapeutic index (TI).

Essential oils	Concentrations in µg/mL (95% confidence interval )		TI (CC_50_/EC_50_)
	EC_50_	CC_50_	
*Zingiber officinale*	33.7 (20.9–55.3)	38.6 (22.3–70.2)	1.12
*Eucalipto globulus*	12.9 (6.4–25.3)	23.9 (15.2–37.7)	1.85
*Thymus vulgaris*	3.1 (1.3–7.4)	2.0 (1.5–2.8)	0.67
“*Illicium verum”*	0.5 (0.3–0.8)	29.5 (17.7–51.7)	60.00
*Rosmarinus officinalis*	57.1 (35.2–93.3)	81.1 (43.2–169.0)	1.42
*Melaleuca alternifolia*	29.2 (18.6–46.7)	41.0 (27.9–60.9)	1.41
*Syzygium aromaticum*	0.09 (0.07–0.13)	0.42 (0.30–0.58)	4.44
*Cymbopogon citratus*	0.11 (0.07–0.19)	0.20 (0.12–0.22)	2.00
*Origanum vulgare*	0.36 (0.23–0.57)	0.41 (0.31–0.56)	1.00
*Citrus limon*	0.15 (0.09–0.22)	1.3 (0.7–2.3)	8.67
*Pelargonium graveolens*	0.20 (0.14–0.29)	1.7 (1.1–2.6)	8.50
*Matricaria recutita*	1.1 (0.6–1.9)	1.2 (0.8–1.7)	1.09

The antiviral activity of drugs against SARS-CoV-2 is widely documented in the literature. For example, in Vero E6 cells, hydroxychloroquine and chloroquine showed an EC_50_ of 1.5 µg/mL and 2.1 µg/mL, respectively^30^. In the study of Pasquereau et al.^31^, chloroquine showed an EC_50_ of 5 µg/mL, while lopinavir exhibited an EC_50_ of 8.81 µg/mL in MRC5 cells. Regarding artemisinin and arbidol, the EC_50_ values were 64.45 µg/mL and 10.70 µg/mL in Vero E6 cells, respectively^32,33^. Therefore, our results indicate that most of the EOs evaluated in this study had similar or better EC_50_ values than the main antiviral drugs already used for the COVID-19 treatment, especially those EOs showing EC_50_ values close or below 1 µg/mL (*S. aromaticum*, *C. citratus*, *C. limon*, *P. graveolens*, *O. vulgare*, “*I. verum”*, and *M. recutita*). It is worth noting that among the diverse cell lines used in pseudoviral assays, HeLa-ACE2 cells proved to be more sensitive in detecting replicating viruses and provided more reproducible titers^34,35^, justifying the choice of the cell line used in our studies.

EOs are well-known for their broad biological spectrum and antiviral potential^18^. Nevertheless, studies evaluating in vitro antiviral activity of EOs against SARS-CoV-2 are very sparse in the literature. The antiviral mechanisms of EOs are still poorly understood, but it is already known that their activity depends mainly on EO compounds and their interactions and some inherent factors concerning the virus, such as the viral load kinetic and viral protein structure. Based on that, the antiviral mechanisms of EOs can be classified into three main ones: target sites during the viral lifecycle (intracellular, intercellular or multiple), morphological alteration (binding to or masking viral structures or destroying them), and protein inhibition through a hydrogen bond, hydrophobic or ionic interactions among others^16,18^. However, it is not yet entirely known for sub-family coronavirinae.

**Figure 1:**
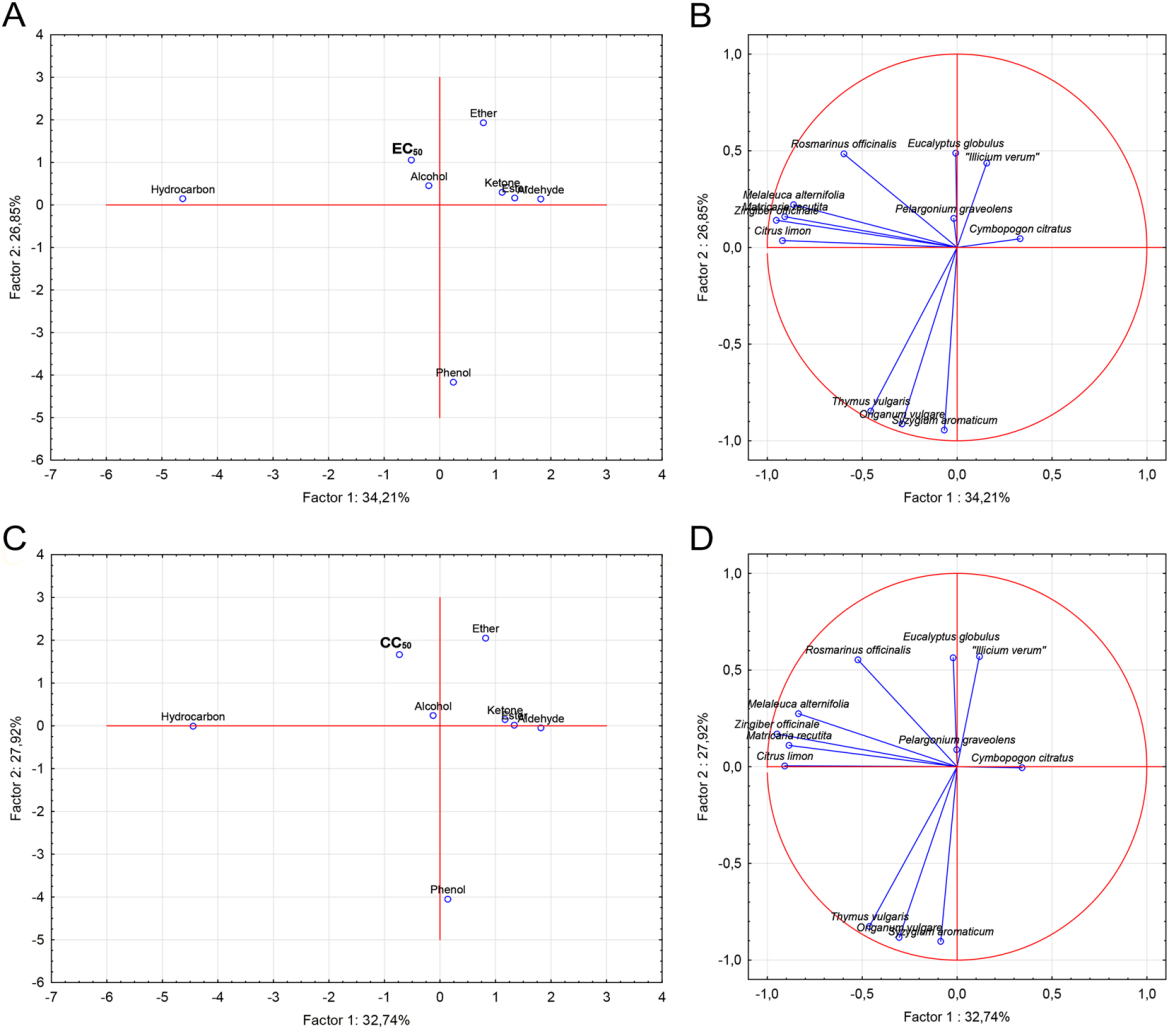
Principal component analysis (PCA) of the functional groups (ketone, alcohol, phenol, aldehyde, hydrocarbon, ether, and ester) within the essential oils regarding the EC_50_ (**A**, **B**) and CC_50_ (**C**, **D**) values with 95% confidence interval.

**Figure 2:**
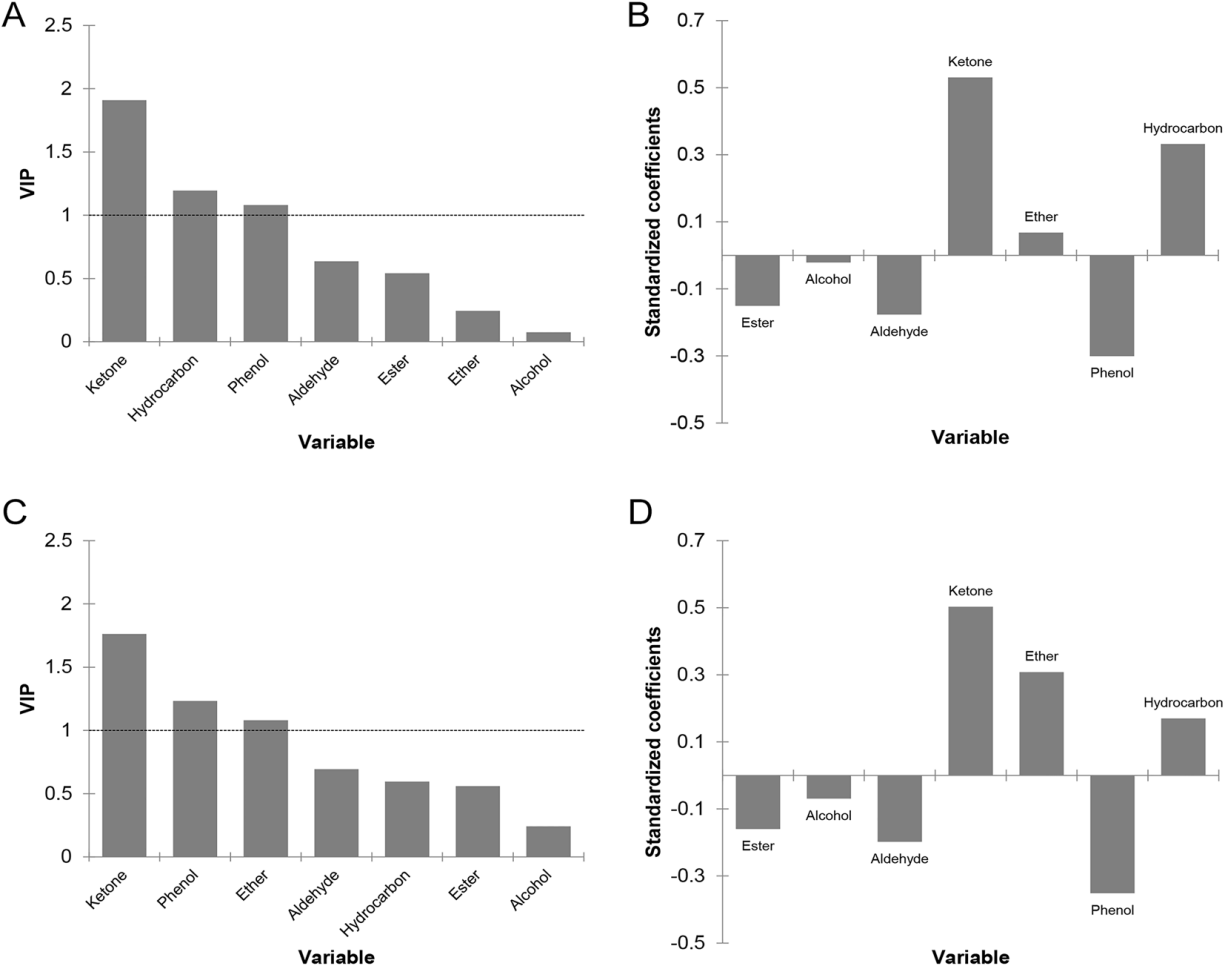
VIP values and standardized coefficients of the functional groups (ketone, alcohol, phenol, aldehyde, hydrocarbon, ether, and ester) within essential oils from partial least-squares regression (PLSR) analysis regarding EC_50_ (**A**, **B**) and CC_50_ (**C**, **D**) values with 95% confidence interval.

Through PCA analysis, the influence of the majority functional groups within each EO on EC_50_ and CC_50_ values can be better understood. Concerning EC_50_ values, PCA 1 and PCA 2 explained 34.21% and 26.85% of the total variance, respectively, and separated the functional groups into three categories: hydrocarbons and alcohol (category 1), phenols (category 2), ethers, aldehydes, esters and ketones (category 3; Fig. 1A). Among these groups, ketones (VIP: 1.910), hydrocarbons (VIP: 1.195) and phenols (VIP: 1.079) had VIP values above 1, indicating a more significant influence on the EC_50_ values (Fig. 2A). However, both ketones and hydrocarbons contributed to an increase in EC_50_ values and, consequently, to a decreased anti-SARS-CoV-2 activity, whereas phenols contributed to a decreased in EC_50_ values and, to an increased antiviral activity (Fig. 2B). Likewise, EC_50_ values were not affected by ether (VIP: 0.243; Fig. 2A), but it revealed a trend decrease in antiviral activity (Fig. 2B). In contrast, although aldehydes, esters, and alcohols have shown no influence on the EC_50_ values (VIP from 0.076 to 0.635; Fig. 2A), they exhibited a potential to decrease EC_50_ values or increase the anti-SARS-CoV-2 activity (Fig. 2B).

There are no reports about the influence of EO functional groups on their antiviral activity. In the present study, most of the EOs had a predominance of hydrocarbons (Supplementary Table 1, Fig. 1A,B), which contributed to a low activity against SARS-CoV-2. A hypothesis is the absence of reactive groups in their structure, such as oxygen or hydroxyls. However, it is worth noting that *C. limon* EO presented 96% of hydrocarbons and showed EC_50_ values of 0.15 µg/mL. It may be attributed to a high concentration of specific hydrocarbons, such as limonene (68.5%) and beta-pinene (10.4%). A previous study reported that limonene compound (73%) was the main responsible for the inhibitory activity of *C. limon* EO against the ACE2 enzyme^29^. Moreover, it is well-known that the EOs may have synergistic or additive interactions due to presenting a broad composition of bioactive compounds. *C. limon* EO was also composed of aldehydes, such as geranial (0.7%) and neral (0.5%), a functional group related to increased antiviral activity against SARS-CoV-2. It also indicates the need for further studies about this EO's composition to understand its activity against ACE2 better. The same was observed for *M. recutita* EO with 56.7% of hydrocarbons, of which 50.6% was (*E*)-beta-farnesene. There are no in vitro reports about the anti-SARS-CoV-2 activity of geranial, neral, and (*E*)-beta-farnesene. However, the last one exhibited a high binding affinity for S_pro_, ACE2, and M_pro_/3CL_pro_^24^, emphasizing the need for further in vitro studies to better understand the individual activity of the compounds. Within alcohol, citronellol (41.5%) was mainly observed in *P. graveolens* (EC_50_: 0.2 µg/mL) (Supplementary Table 1, Fig. 1A, B). A promising antiviral activity was observed for citronellol through binding affinity to the receptor-binding domain (RBD)^36^. Despite that, our findings from PLSR suggest a weak potential anti-SARS-CoV-2 activity by alcohols (Fig. 2A,B). Additionally, this functional group was found as secondary compounds in other EOs with discrepant EC_50_ values as *M. alternifolia* (40.7%; EC_50_: 29 µg/mL) and *M. recutita* (24.1%; EC_50_: 1.1 µg/mL) (Table 1, Supplementary Table 1), reinforcing its low influence on antiviral activity.

The *S. aromaticum*, *T. vulgaris* and *O. vulgare* EOs presented mainly eugenol, carvacrol and/or thymol (Supplementary Table 1, Fig. 1A,B), respectively, which are phenols widely known for their antiviral potential^16^. There are no in vitro studies related to *S. aromaticum* or eugenol, however, this compound was already shown to have binding affinity to to RBD, Mpro/3CLpro, Spro, and ACE2^37–39^. Regarding anti-SARS-CoV-2 in vitro action, *O. vulgare* EO presented a lower EC_50_ value (0.4 µg/mL) than *T. vulgaris* EO (3 µg/mL). This may be due to the higher amount of phenols in *O. vulgare* EO (70.3%) than *T. vulgaris* (56.7%), which were compounds that tended to increase antiviral activity (Fig. 2B). Moreover, *T. vulgaris* EO showed 25.5% of carvacrol, a lower concentration than *O. vulgare* EO (70.3%). According to Ćavar Zeljković et al.^12^, carvacrol alone exhibited activity against SARS-CoV-2, while it was not observed for thymol.

Concerning aldehydes, esters, ketones and ethers categories, the first two showed a tendency to increase the anti-SARS-CoV-2 activity, while the last two demonstrated an opposite trend (Figs. 1A, 2B). The aldehydes geranial and neral were identified as major compounds in *C. citratus* EO (EC_50_ of 0.1 µg/mL) at 45.5% and 33.7%, respectively (Supplementary Table 1, Fig. 1A,B). There are no in vitro reports concerning the activity of this EO against SARS-CoV-2. Nevertheless, geranial and neral are reported to have high in silico affinity for the main protease and for ACE2 (Thuy et al.^40^), corroborating our in vitro results. Furthermore, it is reported that these compounds have a similar mechanism to drugs already known (e.g., hydroxychloroquine and chloroquine) interfering with glycosylation of the SARS-CoV-2 viral spike protein to its receptor (ACE2)^30,41^. It reinforces our findings regarding the tendency of aldehydes, mainly geranial and neral, to increase the antiviral activity of EOs against SARS-CoV-2.

The ester category was represented by *P. graveolens*, *C. citratus*, and *R. officinalis* EOs, which showed this functional group as secondary compounds in their composition (20.7%, 1.7%, and 1.1%, respectively; Supplementary Table 1). Also, *P. graveolens* EO had the widest variety of ester compounds, followed by *C. citratus* and *R. officinalis* EOs. Although they have not been the majority compounds of these EOs, it is suggested that the amount and diversity of esters may have influenced the EC_50_ values. This is because, among three EOs within the ester category, *R. officinalis* showed the lowest concentration and the poorest variety of these compounds, and the lowest antiviral activity (EC_50_: 57 µg/mL). Asif et al.^23^ report a high binding affinity of ester geranyl formate against S_pro_. Additionally, esters have been reported to potentially reverse symptoms related to COVID-19^42^. Despite this, there is very little data in the literature regarding the anti-SARS-CoV-2 action of esters within the EOs, including in vitro studies. The *R. officinalis* EO also presented the highest concentration of ketones (17.3%), emphasizing that it contributed to a lowered anti-SARS-CoV-2 activity (Table 1, Supplementary Table 1, Fig. 2A,B).

The ether category was represented by *E. globulus* (EC_50_: 13 µg/mL) and labeled as *I. verum* (EC_50_: 0.5 µg/mL), which presented mostly1,8-cineole (86.2%) and (*E*)-anethole (90.7%), respectively (Supplementary Table 1, Fig. 1A,B). Although in silico studies have revealed some affinity of the 1,8-cineole for S_pro_^37^, RBD^28,43^, M_pro_/3CL_pro_^44^, and TMPRSS2^43^, its in vitro activity against SARS-CoV-2 in Vero 76 cells was very low (EC_50_ = 835.85 µg/mL)^12^. Furthermore, *E. globulus* had a reasonable amount of hydrocarbons (13.9%), which may explain the contrasting EC_50_ values compared to “*I. verum”*, two EOs containing mostly ether grouped within the same PCA category. This fact associated with our findings on ether tending to decrease the anti-SARS-CoV-2 activity (Fig. 2B) may justify the high EC_50_ values for *E. globulus*. Otherwise, in vitro studies evaluating antiviral activity from compound (*E*)-anethole has not yet been reported. Therefore, (*E*)-anethole seems to be a specific ether compound with potential activity against SARS-CoV-2 when predominantly present in an EO (about or above 90%). It is worth highlighting that 9% of triacetin (a not natural compound) was found in product labeled as “*I. verum”*. However, it did not demonstrate in vitro anti- SARS-CoV-2 activity (EC_50_: 2400 µg/mL), indicating that (*E*)-anethole was indeed responsible for the antiviral activity.

Aside from antiviral activity, the cell viability study is an important parameter to ensure that effective EOs against viral agents do not exert toxicity on the human host cells^16,45^. In the PCA analysis concerning CC_50_ values (Fig. 1C,D), the first and the second components explained 32.74% and 27.92% of the total variance, respectively, and separated the functional group treatments into three categories, likewise for EC_50_ values: hydrocarbons and alcohols (category 1), phenols (category 2), and aldehydes, ethers, esters and ketones (category 3; Fig. 1C). Among these groups, ketones (VIP: 1.763), phenols (VIP: 1.232) and ether (VIP: 1.080) had VIP values above 1, indicating a more significant influence on the CC_50_ values (Fig. 2C). In Fig. 2D, it was observed that ketones and ether contributed to an increase in CC_50_ values and, consequently, to a decreased cytotoxicity, however, the presence of phenols contributed to increase the cytotoxicity. In addition, despite hydrocarbons having a VIP value of 0.596, this functional group also showed a trend of reduced cytotoxicity (Fig. 2C,D). Likewise, CC_50_ values were not affected by alcohols (VIP: 0.243), esters (VIP: 0.560), and aldehydes (VIP: 0.694; Fig. 2C), but they revealed a trend increasing in cytotoxicity (Fig. 2D).

Based on Fig. 1C,D, it is possible to observe that hydrocarbons also strongly influenced the grouping of the EOs considering CC_50_ values. The influence of the composition of EOs is complex, where each compound acts individually and/or interacts^46^. Furthermore, the compound cytotoxicity can be based on mechanisms such as cell death by apoptosis and/or necrosis, cell cycle arrest, loss of key organelles function, and damage of cell membranes, causing reduced production of ATP, pH gradient changes, and loss of mitochondrial potential, among others^46–48^. In this context, the cytotoxic activity of EOs may be related to properties of mono and sesquiterpenes, such as lipophilicity and molecular weight, justifying PCA grouping.

The EOs showed CC_50_ values ranging from 0.2 to 81 µg/mL (Table 1). Overall, functional groups that increased or tended to increase EC_50_ values also increased or tended to increase CC_50_ values (Fig. 2C,D). In other words, functional groups that contributed to a decreased activity against SARS-CoV-2 were those that contributed to a decreased cytotoxicity for human cells. It may be clearly noted in our findings. *Z. officinale*, *M. alternifolia*, and *R. officinalis* presented the lowest cytotoxicity but also the lowest anti-SARS-CoV-2 activity (Table 1). Likewise, EOs exhibiting the highest antiviral activity were the most cytotoxic (*S. aromaticum*, *C. citratus*, *C. limon*, *P. graveolens*, *O. vulgare*, and *M. recutita*), except the “*I. verum”*. This EO demonstrated effectiveness against SARS-CoV-2 and low cytotoxicity (Table 1), suggesting the great potential of the (*E*)-anethole alone (without the influence of other EO compounds) to block cellular entry of SARS-CoV-2 with no human cell damage.

Among the EOs with higher cytotoxicity, those that showed the highest ones were *C. citratus, O. vulgare,* and *S. aromaticum*. Like antiviral activity, the EOs cytotoxicity is attributed to their major chemical constituents^16^. *C. citratus* EO was mainly composed of geranial and neral compounds (aldehydes), *O. vulgare* EO of carvacrol (phenol), and *S. aromaticum* EO of eugenol (phenol). All of these compounds have already shown high cytotoxicity in HeLa cells in previous studies^49,50^, corroborating our findings. However, it is worth mentioning that more studies need to be carried out to understand the cytotoxic activity of EOs, including evaluating secondary compounds present in their composition and their influence on anti-SARS-CoV-2 activity.

Regarding the therapeutic index (TI), this parameter depends on the EC_50_ and CC_50_ values aiming exactly to attain EOs with higher antiviral activity and lower cytotoxicity. Stránská et al.^51^ reported that a TI greater than 4 is satisfactory for natural products. In this way, the EOs that presented acceptable values of TI in descending order were “*I. verum”* (TI: 60), *C. limon* (TI: 8.67), *P. graveolens* (TI: 8.50), and *S. aromaticum* (TI: 4.44)*.* The low cytotoxicity in HT-29 cells of *C. limon* and *P. graveolens* EOs has already been reported in the literature by Senthil Kumar et al.^29^, which also suggested that both EOs may impair the viral cellular entry during SARS-CoV-2 infection through an anti-ACE2 activity in vitro assay. Nevertheless, these authors did not determine TI values. Regarding the product labeled as “*I. verum”,* composed of ((E)-anetole compound) and triacetin, and *S. aromaticum* EOs, no study has yet reported neither anti-SARS-CoV-2 activity or TI values. The contaminant triacetin present in “*I. verum”* composition showed EC_50_ of 2400 µg/mL, CC_50_ of 1600 µg/mL, and TI of 0.66. Therefore, the antiviral and cytotoxicity activities of this EO were due to its main compound ((*E*)-anethole), which was responsible for the highest value of TI in our study, revealing its potential against SARS-CoV-2. The high TI found for *C. limon* EO may be attributed to potential specific hydrocarbons (limonene and beta-pinene), and their interaction with other compounds from another functional group, such as geranial and neral (aldehydes), wherein all of them conferred better anti-SARS-CoV-2 activity to EOs. *P. graveolens* and *S. aromaticum* presented a predominance of citronellol and eugenol, respectively, that contributed to strongly reducing EC_50_ values and obtaining good TI values.

The activity of functional groups, specific compounds, and their interactions directly influenced the anti-SARS-CoV-2 and HeLa cells' cytotoxicity actions and, consequently, the TI values. The functional groups able to improve activity against SARS-CoV-2 also increased the cytotoxicity of the EOs. Hydrocarbons, ketones, and ethers decreased or tended to decrease the anti-SARS-CoV-2 activity and cytotoxicity, while the opposite was observed for aldehydes, phenols, alcohols, and esters. Some specific compounds, such as (*E*)-anetole, eugenol, limonene, beta-pinene, and citronellol, can be highlighted due to their majority presence in EOs with greater activity against the SARS-CoV-2 delta variant. From interactions, product labeled as “*I. verum”*, *C. limon*, *P. graveolens,* and *S. aromaticum* were the EOs most effective against SARS-CoV-2 while minimizing cytotoxicity (TI above 4). In this context, they could be helpful in different ways, such as developing new drugs, including the use of nanoencapsulation, or using their volatile characteristics through spray formulation for the nasal and oral cavities to suppress COVID-19 infection^52^. However, more in vitro studies are needed to understand the compound interactions in these four potential EOs leading to great TI against SARS-CoV-2 and its variants.

## Methods

### Plant material

The essential oils of *Zingiber officinale*, *Eucalyptus globulus*, *Thymus vulgaris*, *Rosmarinus officinalis*, *Melaleuca alternifolia*, *Syzygium aromaticum*, *Cymbopogon citratus*, *Origanum vulgare*, *Citrus limon*, *Pelargonium graveolens*, *Matricaria recutita* and product labeled as *Illicium verum* (see Supplementary Table 1) were acquired from Quinari (Quinari—Óleos e essências, Paraná, Brazil). According to the manufacturer, the parts used for extracting the EOs were the leaves (*C. citratus, E. globulus, R. officinalis, M. alternifolia, O. vulgare,* and *T. vulgaris*), seeds (*I. verum*), rhizome (*Z. officinale*), shells (*C. limon*), flowers (*M. recutita*), seeds and leaves (*S. aromaticum*), and flowers and leaves (*P. graveolens*). Triacetin (99%) was purchased from the Sigma Aldrich ^®^ (St. Louis, MO).

### Characterization of EOs by GC–MS and GC-FID

The composition of EOs was determined using gas chromatography coupled to mass spectrometry (GC–MS). The analyses were performed in an Agilent 7890A gas chromatography coupled to a 5975C mass detector and equipped with a 5% diphenyl–95% dimethylpolysiloxane capillary column (DB-5 MS, 30 m × 0.25 mm × 0.25 µm). The oven temperature was programmed to rise from 60 to 240 °C at 3 °C/min. The carrier gas was helium at 1.0 mL/min. Each pure oil was dissolved in hexane (0.1%), and 1.0 µL was injected through an Agilent 7693A autosampler in split mode (1:50) with the injector at 250 °C. The transfer line was kept at 260 °C, the ion source at 230 °C, and the analyzer at 150 °C. The mass detector was operated in electron ionization mode (70 eV), with 3.15 scans/second, and data was collected in the 40–350 m/*z* range. The EO compounds were identified by comparing their mass spectra with those found in the Wiley Registry of Mass Spectral Data^53^ and Adam's library^54^, and their linear retention indices were calculated according to^55^. For quantitation, the samples were injected in an Agilent 7890A gas chromatograph equipped with a flame ionization detector (FID) operating at 280 °C, using the same column and analytical conditions as described for GC–MS, except for the carrier gas, which, in this case, was hydrogen (1.5 mL/min). An internal standard (methyl octanoate) was added to all samples. The raw peak areas were normalized using the internal standard area^56^ and corrected using predicted response factors^57^. All calculations were performed using a series of pre-programmed Excel electronic sheets^58^. The FID analyses were carried out in triplicate.

### Pseudovirus (PsV) production and cell line

The HeLa cells expressing human angiotensin-converting enzyme 2 (HeLa ACE-2) were provided by Dr. Dennis Burton (The Scripps Research Institute, La Jolla, CA). SARS-CoV-2 pseudovirus was produced from plasmids containing the SARS-CoV-2 spike genes [pSARS-CoV2-Strunc delta, pCRV1NHG GagPol, and pNanoLuc2AEGFP] following procedures described by Schmidt et al.^59^ and modified by Alsaidi et al.^60^.

#### Antiviral and cytotoxicity assays

The antiviral and cytotoxicity activities were determined using the cell-based pseudoviral entry and the XTT assay, respectively^60^. All samples were first diluted in complete medium (Dulbecco’s Modified Eagles Medium, 10% FBS, 1% Penicillin + Streptomycin, Thermofisher Scientific, Waltham, MA) containing 20% Dimethyl sulfoxide (Sigma Aldrich, St. Louis, MO). From this stock, nine different dilutions were prepared in the complete medium. For *Z. officinale*, *E. globulus*, *T. vulgaris*, *I. verum*, *R. officinalis*, and *M. alternifolia*, the dilutions ranged from 10,000 to 1.52 µg/mL and for *S. aromaticum*, *C. citratus*, *O. vulgare*, *C. limon*, *P. graveolens*, and *M. recutita* from 500 to 0.08 µg/mL. For cytotoxicity assay, these different dilutions were added in triplicates to clear bottom 96-well microplates containing HeLa ACE-2 cells, and it was incubated at 37 °C, 5% CO_2_, and 98% humidity for 72 h. XTT (ThermoFisher Scientific, Waltham, MA) was added to all wells after 72-h incubation, and the absorbance was measured at 450 nm using a Spectramax iD3 (Molecular Devices, San Jose, CA). For the antiviral assay, different dilutions of each sample were pre-incubated in triplicates with SARS-CoV-2 Delta PsV at 37 °C, 5% CO_2_, and 98% humidity for 30 min at 37 °C, 5% CO_2_, and 98% humidity. The mixture was then transferred to 96-well white opaque microplates containing HeLa ACE-2 cells and the plates were incubated under the same conditions previously mentioned. The TurboLuc™ Luciferase One-Step Glow Assay Kit (ThermoFisher Scientific) was used to determine the percentage entry of PsV in each sample dilution versus the virus control. The half-maximal effective concentration (EC_50_), half-maximal cytotoxic concentration (CC_50_), and therapeutic index (TI = CC_50_/EC_50_) were determined for each sample.

### Data analysis and chemometrics approach

For EC_50_ and CC_50_, all samples' dilutions were tested in triplicate in each independent experiment (*n* = 2). For dose–response–inhibition analysis, the GraphPad Prism software was used (version 9.0.2, GraphPad Sofware, San Diego, California, USA). The principal component analysis (PCA) was used to identify associations between EOs and their composition with EC_50_ and CC_50_ values on Statistica 10.0 software (StatSoft, Oklahoma, USA). The partial least-squares regression (PLSR) was applied to determine which functional groups were determinants to increase or decrease the EC_50_ and CC_50_ values, considering the partial least square standardized coefficients and the variable importance in projection (VIP) > 1. For PLSR analysis, it was used the XLSTAT software, version 2021.1 (Addinsoft, New York, New York, USA). All statistical analyses were carried out at a 5% significance level.

## Supplementary Information

Supplementary Information.

## Data Availability

The data that support the findings of this study are available from the corresponding author upon reasonable request.
