# Supplementary material for: Essential oils block cellular entry of SARS-CoV-2 delta variant
Source: Sci Rep. 2022 Nov 30;12:20639. doi: 10.1038/s41598-022-25342-8 (PMC9709744; doi:10.1038/s41598-022-25342-8)
Supplement: Supplementary file 1 — Supplementary Information. [file 41598_2022_25342_MOESM1_ESM.pdf]

Supplementary Table 1. Composition of twelve essential oils through gas chromatography coupled to mass spectrometry (GC-MS) and gas chromatography coupled to flame ionization detector (CG-FID).

Illicium verum[*]				
Peak	RI exp[¥]	RI lit[€]	Identification	Area %**
1	1287	1282	(E)-anetole	90.7
2	1356	1247	triacetin	9.3
Total:				-
Total identified:				-
Monoterpenes (%):				-
Oxygenated monoterpenoids (%):				-
Sesquiterpenes (%):				-
Oxygenated sesquiterpenoids (%):				-
Others (%):				-

Syzygium aromaticum				
Peak	RI exp	RI lit	Identification	Area %
1	1361	1356	eugenol	87.9
2	1413	1417	(E)-beta-caryophyllene	9.0
3	1446	1452	alpha-humulene	1.9
4	1517	1423	delta-cadinene	0.3
5	1545	-	n.i.	0.2
6	1575	1582	caryophyllene oxide	0.7
Total:				100.0
Total identified:				100.0
Monoterpenes (%):				0
Oxygenated monoterpenoids (%):				0
Sesquiterpenes (%):				11.2
Oxygenated sesquiterpenoids (%):				0.9
Others (%):				87.9

Thymus vulgaris				
Peak	RI exp	RI lit	Identification	Area %
1	930	932	alpha-pinene	3.0
2	944	946	camphene	2.2
3	988	988	mircene	1.3
4	1021	1022	p-cymene	21.7
5	1025	1024	limonene	3.4
6	1028	1026	1,8-cineole	2.5
7	1099	1098	linalool	6.0
8	1162	1165	borneol	3.4
9	1291	1289	thymol	31.2
10	1301	1298	carvacrol	25.5
Total:				100.0
Total identified:				100.0
Monoterpenes (%):				31.5
Oxygenated monoterpenoids (%):				68.5
Sesquiterpenes (%):				-
Oxygenated sesquiterpenoids (%):				-
Others (%):				-

Eucalyptus globulus				
Peak	RI exp	RI lit	Identification	Area %
1	930	932	alpha-pinene	2.0
2	973	974	beta-pinene	0.4
3	988	988	myrcene	0.7
4	1003	1002	alpha-phelandrene	0.6
5	1014	1014	alpha-terpinene	0.2
6	1022	1022	p-cymene	3.3
7	1026	1024	limonene	4.9
8	1030	1026	1,8-cineole	86.2
9	1034	1032	(Z)-beta-ocymene	0.2
10	1044	1044	(E)-beta-ocymene	0.1
11	1054	1054	gamma-terpinene	1.5
Total:				100.0
Total identified:				100.0
Monoterpenes (%):				13.8
Oxygenated monoterpenoids (%):				86.2
Sesquiterpenes (%):				-
Oxygenated sesquiterpenoids (%):				-
Others (%):				-

Cymbopogon citratus				
Peak	RI exp	RI lit	Identification	Area %
1	920	921	tricyclene	tr

Origanum vulgare				
Peak	RI exp	RI lit	Identification	Area %
1	920	921	tricyclene	0.1

Citrus limon				
Peak	RI exp	RI lit	Identification	Area %
1	924	924	alpha-tujene	0.4

Matricaria recutita				
Peak	RI exp	RI lit	Identification	Area %
1	1058	1056	3,3,6-trimetil-1,5-heptadien-4-one	0.1

2	930	932	alpha-pinene	0.1
3	945	946	camphene	0.3
4	987	981	6-methyl-5-hepten-2-one	0.4
5	990	988	dehydro-1,8-cineole	0.1
6	1025	1024	limonene	0.9
7	1034	1032	(Z)-beta-ocymene	0.4
8	1044	1044	(E)-beta-ocymene	0.2
9	1070	-	4-nonanone	0.3
10	1099	1098	linalool	0.6
11	1103	-	n.i.	0.1
12	1142	-	n.i.	0.6
13	1146	-	n.i.	0.2
14	1150	1148	citronellal	1.3
15	1162	-	n.i.	1.8
16	1180	-	n.i.	2.6
17	1227	1223	citronellol	1.1
18	1237	1235	neral	33.7
19	1253	1249	geraniol	4.5
20	1266	1264	geranial	45.5
21	1351	1350	citronellyl acetate	0.2
22	1381	1379	geranyl acetate	1.5
23	1386	1389	beta-elemene	0.4
24	1411	1417	(E)-beta-caryophyllene	0.9
25	1445	1452	alpha-humulene	0.1
26	1473	1480	germacrene D	0.2
27	1506	1513	gamma-cadinene	0.4
28	1516	1522	delta-cadinene	0.3
29	1543	1548	elemol	0.9
30	1575	1572	caryophyllene oxide	0.5

2	924	924	alpha-tujene	0.0
3	930	932	alpha-pinene	1.2
4	945	946	camphene	0.4
5	973	974	beta-pinene	0.3
6	977	974	1-octen-3-ol	0.1
7	980	-	n.i.	0.1
8	988	988	myrcene	1.6
9	1003	1002	alpha-phelandrene	0.2
10	1008	1008	delta-3-carene	0.1
11	1014	1014	alpha-terpinene	1.0
12	1019	1020	m-cymene	0.1
13	1022	1022	p-cymene	10.4
14	1025	1024	limonene	0.6
15	1028	1026	1,8-cineole	0.5
16	1054	1054	gamma-terpinene	4.8
17	1099	1098	linalool	2.2
18	1162	1165	borneol	0.2
19	1174	1174	terpinen-4-ol	0.4
20	1292	1289	thymol	0.4
21	1300	1298	carvacrol	70.3
22	1412	1417	(E)-beta-caryophyllene	4.7
23	1445	1452	alpha-humulene	0.1
24	1575	1582	caryophyllene oxide	0.3

2	930	932	alpha-pinene	1.8
3	945	946	camphene	0.1
4	970	969	sabinene	1.5
5	974	974	beta-pinene	10.4
6	988	988	myrcene	1.5
7	1004	1002	alpha-phelandrene	0.1
8	1014	1014	alpha-terpinene	0.2
9	1022	1022	p-cymene	1.0
10	1027	1024	limonene	68.5
11	1029	1026	1,8-cineole	0.4
12	1044	1044	(E)-beta-ocymene	0.1
13	1055	1054	gamma-terpinene	9.3
14	1085	1086	terpinolene	0.4
15	1100	1098	linalool	0.2
16	1174	1174	terpinen-4-ol	0.3
17	1188	1186	alpha-terpineol	0.5
18	1239	1235	neral	0.6
19	1268	1264	geranial	0.8
20	1362	1359	neryl acetate	0.5
21	1411	1417	(E)-beta-caryophyllene	0.2
22	1429	1432	(E)-alpha-bergamotene	0.5
23	1502	1505	beta-bisabolene	0.8

2	1372	1374	alpha-copaene	0.3
3	1379	1387	alpha-isocomene	0.2
4	1386	1389	beta-elemene	0.1
5	1411	1417	(E)-beta-caryophyllene	0.4
6	1451	1454	(E)-beta-farnesene	50.6
7	1464	-	n.i.	1.9
8	1474	1480	germacrene D	1.0
9	1479	1489	beta-selinene	0.4
10	1489	-	n.i.	0.6
11	1503	1505	(E,E)-alpha-farnesene	1.5
12	1517	1523	delta-cadinene	0.2
13	1559	-	n.i.	0.4
14	1570	1577	spathulenol	2.6
15	1586	1594	salvial-4(14)-en-1-one	0.1
16	1605	-	n.i.	0.3
17	1610	-	n.i.	0.4
18	1620	-	n.i.	0.2
19	1624	-	n.i.	0.7
20	1633	1638	gossonorol + epi-alpha-cadinol	1.1
21	1649	1656	alpha-bisabolol oxide B	8.5
22	1655	-	n.i.	1.3
23	1663	-	n.i.	0.2
24	1678	1685	alpha-bisabolol	5.4
25	1682	-	n.i.	0.6
26	1721	1730	chamazulene	2.0
27	1740	1748	alpha-bisabolol oxide A	6.2
28	1751	-	n.i.	0.2
29	1794	-	n.i.	0.1
30	1841	-	n.i.	0.2

															31	1878	1879	(Z)-chamomile spiroether	10.4
															32	1892	1890	(E)-chamomile spiroether	0.8
															33	1946	-	n.i.	0.4
															34	2135	2135	linoleic acid	0.2
															35	2492	2500	pentacosane	0.3
Total:				100.0					100.0					100.0					100.0
Total identified:				94.8					99.9					100.0					92.4
Monoterpenes :				1.9					20.7					95.3					0.0
Oxygenated monoterpenoids:				93.7					74.0					3.2					0.1
Sesquiterpenes:				2.3					4.8					1.5					55.2
Oxygenated sesquiterpenoids:				1.5					0.3					0.0					32.0
Others:				0.6					0.1					0.0					12.7

Rosmarinus officinalis					*Pelargonium graveolens*					*Zingiber officinale*					*Melaleuca alternifolia*				
Peak	RI exp	RI lit	Identification	Area %	Peak	RI exp	RI lit	Identification	Area %	Peak	RI exp	RI lit	Identification	Area %	Peak	RI exp	RI lit	Identification	Area %
1	918	-	n.i.	tr	1	930	932	alpha-pinene	0.2	1	920	921	tricyclene	0.4	1	924	924	alpha-tujene	1.0
2	921	921	tricyclene	0.7	2	988	988	myrcene	0.1	2	930	932	alpha-pinene	2.1	2	931	932	alpha-pinene	2.5
3	925	924	alpha-tujene	0.1	3	1025	1024	limonene	0.1	3	944	946	camphene	9.3	3	970	969	sabinene	1.3
4	931	932	alpha-pinene	15.3	4	1070	1067	cis-linalool oxide	0.1	4	970	969	sabinene	0.1	4	974	974	beta-pinene	0.8
5	946	946	camphene	6.5	5	1099	1098	linalool	7.8	5	973	974	beta-pinene	0.3	5	989	988	myrcene	0.8
6	951	-	n.i.	0.2	6	1109	1106	cis-rose oxide	2.0	6	986	981	6-methyl-5-hepten-2-ona	0.3	6	1004	1002	alpha-phelandrene	0.5
7	975	974	beta-pinene	5.1	7	1125	1122	trans-rose oxide	1.0	7	988	988	myrcene	1.2	7	1015	1014	alpha-terpinene	10.3
8	989	988	myrcene	4.9	8	1150	1148	mentone	2.4	8	1003	1002	alpha-phelandrene	0.4	8	1022	1022	p-cymene	1.8
9	1004	1002	alpha-phelandrene	0.4	9	1160	1158	isomenthone	6.2	9	1021	1022	p-cymene	0.1	9	1026	1025	beta-phelandrene	1.9
10	1009	1008	delta-3-carene	0.1	10	1188	1186	alpha-terpineol	0.2	10	1025	1025	beta-phelandrene	5.8	10	1028	1026	1,8-cineole	2.6
11	1015	1014	alpha-terpinene	0.3	11	1229	1223	citronellol	41.5	11	1028	1026	1,8-cineole	3.3	11	1056	1054	gamma-terpinene	20.6
12	1022	1022	p-cymene	2.0	12	1253	1249	geraniol	9.6	12	1085	1086	terpinolene	0.2	12	1065	1065	cis-sabinene hydrate	0.2

13	1026	1024	limonene	4.6
14	1030	1026	1,8-cineole	31.7
15	1055	1054	gamma-terpinene	0.9
16	1086	1086	terpinolene	0.4
17	1100	1098	linalool	1.0
18	1142	1141	camphor	16.6
19	1154	1155	isoborneol	0.6
20	1163	1165	borneol	1.8
21	1175	1174	terpinen-4-ol	0.5
22	1189	1186	alpha-terpineol	1.5
23	1196	1199	gamma-terpineol	0.2
24	1208	1204	verbenone	0.7
25	1283	1283	isobornyl acetate	1.1
26	1412	1417	(E)-beta-caryophyllene	2.6
27	1446	1452	alpha-humulene	0.2

13	1269	1264	geranial	0.2
14	1273	1271	citronellila formate	12.4
15	1300	1298	geranyl formate	2.7
16	1351	1350	citronellyl acetate	0.5
17	1369	1374	alpha-copaene	0.2
18	1378	1387	beta-bourbonene	0.6
19	1386	1390	beta-elemene	0.1
20	1411	1417	(E)-beta-caryophyllene	0.8
21	1432	1437	alpha-guayene	0.2
22	1437	1442	6,9-guaiadiene	3.2
23	1440	-	n.i.	0.7
24	1446	1452	alpha-humulene	0.2
25	1453	1458	allo-aromadendrene	0.2
26	1466	1465	cis-muurola-4(14),5-diene	0.1
27	1473	1476	geranyl propionate	1.1
28	1488	1496	viridiflorene	0.6
29	1507	1513	gamma-cadinene	0.1
30	1517	1522	delta-cadinene	0.7
31	1525	1530	citronell butanoate	0.8
32	1557	1562	geranyl butanoate	0.7
33	1582	1584	2-phenyl-ethyl tiglate	0.8
34	1612	1622	10-epi-gamma-eudesmol	0.2
35	1662	1666	citronellyl tiglate	0.5
36	1697	1696	geranyl tiglate	1.2

13	1095	-	n.i.	0.3
14	1099	1098	linalool	0.3
15	1161	1165	borneol	1.4
16	1174	1174	terpinen-4-ol	0.3
17	1188	1186	alpha-terpineol	0.6
18	1332	1335	delta-elemene	0.2
19	1358	-	n.i.	0.2
20	1369	1374	alpha-copaene	0.7
21	1386	1389	beta-elemene	0.9
22	1401	-	n.i.	0.3
23	1412	1417	(E)-beta-caryophyllene	0.1
24	1428	1430	gamma-elemene	0.6
25	1430	1432	(E)-alpha-bergamotene	0.1
26	1453	1458	allo-aromadendrene	1.1
27	1470	1478	gamma-muurolene	0.5
28	1474	1480	germacrene D	1.4
29	1478	1480	ar-curcumene	6.6
30	1486	-	n.i.	1.1
31	1493	1493	alpha-zingiberene	34.9
32	1504	1505	(E,E)-alpha-farnesene	10.6
33	1510	-	n.i.	0.4
34	1520	1521	beta-sesquiphelandrene	11.8
35	1527	1529	(E)-gamma-bisabolene	0.3
36	1544	1548	elemol	0.4
37	1549	1559	germacrene B	0.3
38	1560	1561	(E)-nerolidol	0.3
39	1585	-	n.i.	0.2
40	1609	-	n.i.	0.3

13	1085	1086	terpinolene	3.7
14	1098	1098	trans-sabinene hydrate	0.5
15	1120	1118	cis-p-menth-2-en-1-ol	0.4
16	1137	1136	trans-p-menth-2-en-1-ol	0.3
17	1176	1174	terpinen-4-ol	35.7
18	1188	1186	alpha-terpineol	2.4
19	1369	1374	alpha-copaene	0.1
20	1402	1409	alpha-gurjunene	0.5
21	1412	1417	(E)-beta-caryophyllene	0.6
22	1431	1439	aromadendrene	1.7
23	1436	1444	seline-5,11-diene	0.2
24	1443	1450	trans-muurola-3,5-diene	0.2
25	1445	1452	alpha-humulene	0.2
26	1453	1460	allo-aromadendrene	0.7
27	1467	1475	trans-cadina-1(6),4-diene	0.3
28	1478	1489	beta-selinene	0.1
29	1480	-	n.i.	0.1
30	1484	1495	cis-cadina-1(6),4-diene	0.3
31	1488	-	n.i.	1.4
32	1489	1494	bicyclogermacrene	1.1
33	1494	1500	alpha-muurolene	0.2
34	1517	1522	delta-cadinene	1.6
35	1518	-	n.i.	0.3
36	1525	1533	trans-cadina-1,4-diene	0.2
37	1552	-	n.i.	0.1
38	1570	1577	spathulenol	0.1
39	1576	-	n.i.	0.8
40	1584	1592	viridiflorol	0.3
41	1586	1595	cubeban-11-ol	0.3

				42	1595	1600	rosifoliol	0.2
				43	1615	-	n.i.	0.3
				44	1621	1629	epi-cubenol	0.4
				45	1631	-	n.i.	0.1
				46	1635	1645	cubenol	0.2
Total:	100.0	100.0	100.0					100.0
Total identified:	99.8	99.3	97.2					96.8
Monoterpenes :	39.6	0.4	19.8					45.2
Oxygenated monoterpenoids:	57.1	71.1	6.2					42.1
Sesquiterpenes:	3.4	7.7	72.4					9.9
Oxygenated sesquiterpenoids:	-	0.2	1.3					2.8
Others:	-	20.7	0.3					0.0

n.i.: Not identified
tr: trace (<0.1%)
[¥]RI exp: Experimental Retention Index;
[€]RI lit: Literature Retention Index (Adams);
*Essential oil labeled as *Illicium verum* containing non-corresponding composition and triacetin. which is not considered a natural compound;
** Normalized and corrected percentage area with response factors.
